# Influenza vaccination knowledge, attitudes, and practices among Tunisian elderly with chronic diseases

**DOI:** 10.1186/s12877-021-02667-z

**Published:** 2021-12-15

**Authors:** Ghassen Kharroubi, Ines Cherif, Leila Bouabid, Adel Gharbi, Aicha Boukthir, Nissaf Ben Alaya, Afif Ben Salah, Jihene Bettaieb

**Affiliations:** 1 Laboratory of Medical Epidemiology, Pasteur Institute of Tunis, 13, Place Pasteur, B.P.74, 1002, Belvédère, Tunis, Tunisia; 2 Laboratory of Transmission, Control and Immunobiology of Infections (LR11IPT02), Pasteur Institute of Tunis, Tunis, Tunisia; 3 National Observatory of New and Emerging Diseases, Tunis, Tunisia; 4 Department of Family and Community Medicine, College of Medicine and Medical Sciences, Arabian Gulf University, Manama, Bahrain

**Keywords:** Older persons, Flu, Vaccination coverage, Vaccination intention, Tunisia

## Abstract

**Background:**

Generally, seasonal influenza does not cause severe infection in healthy adults, but for the elderly, an infection can pose a serious health concern. Although several measures can help prevent influenza, vaccination is considered the most effective. This study aimed to assess influenza vaccine uptake among elderly with chronic diseases in Tunisia during the 2018–2019 influenza season, and to identify knowledge, attitudes and barriers associated with influenza vaccine uptake.

**Methods:**

During influenza season of 2018–2019, we conducted a national cross-sectional study among elderly with chronic disease who were attending primary and secondary health care facilities in Tunisia. We collected data regarding practices, general knowledge and attitudes related to influenza and influenza vaccine, using a standardized questionnaire. A multivariate analysis by logistic regression was performed to assess the factors influencing willingness to receive influenza vaccine.

**Results:**

Among the 1191 surveyed elderly, 19.4% (95%CI 14.1–21.9) were vaccinated during the 2018–2019 influenza season and 64.7% (61.9–67.3) expressed willingness to be vaccinated in the next season regardless of vaccination status in the 2018–2019 season. Previous vaccination in the 2018–2019 influenza season was the most significantly associated factor with willingness to receive influenza vaccine (adjusted OR = 16.5 [3.7–72.4]). Significant associations were also observed between knowledge of influenza severity for the elderly as well as for those with chronic diseases and willingness to be vaccinated (*p* < 0.01). Likewise, participants who were convinced by flu vaccine effectiveness and those who were not concerned about vaccine side effects were more likely to be vaccinated (*p* < 0.001). The main reason that may lead to vaccine acceptance was a doctor’s recommendation (41.1%), while the two main reasons that may lead to vaccine refusal were concerns about side effects (71.5%) and a belief that vaccine was ineffective in averting influenza illness (33.9%). Doctors were the most trusted source for information about influenza vaccine (91.5%).

**Conclusion:**

Our study revealed low influenza vaccination coverage among Tunisian elderly with chronic diseases believed to be at higher risk for severe acute respiratory infections and death if infected with influenza. Treating physicians’ role in promoting influenza vaccination in this high-risk group seems to be crucial.

**Supplementary Information:**

The online version contains supplementary material available at 10.1186/s12877-021-02667-z.

## Introduction

Seasonal influenza is an infection of the airways caused by various influenza viral strains that annually undergo antigenic variations [1, 2]. This illness has a significant impact on both individuals and societies, and is responsible for a considerable economic burden in terms of treatment and hospitalizations [3, 4]. The World Health Organization (WHO) estimates that influenza infects about 5–15% of the population annually. The estimated annual global burden of influenza is nearly 3 to 5 million cases of severe disease, with a death toll ranging from 290,000 and 650,000 persons [5]. Generally, seasonal influenza does not cause severe infection in healthy adults, but for the elderly, an infection can pose a serious health concern [6]. The severity of influenza among the elderly is increased by the higher prevalence of comorbidities among this age group [6].

Although several measures can help prevent influenza, vaccination is considered the most effective [7]. Thus, in 2003, the World Health Assembly (WHA) urged all its Member States to implement strategies in order to increase influenza vaccination coverage of all high-risk individuals, including the elderly and those with underlying diseases, with the goal of reaching a minimum of 75% vaccination coverage by 2010 [4, 8]. Many countries have already implemented policies promoting influenza vaccination for high-risk groups, especially in the older populations [3, 9]. However, vaccine uptake for aging populations still remain below the WHO recommendations, even in developed countries [3]. Vaccine hesitancy, defined as the delay in vaccine acceptance or refusal regardless of vaccine availability [10], was found to have a detrimental impact on vaccine uptake [11]. Some factors influencing vaccine hesitancy were identified in previous studies [12, 13] such as concerns about vaccine efficacy and safety, physician-patient relationship and media environment.

In Tunisia, influenza vaccine is available in public health care facilities and free of charge for elderly patients under practitioners’ prescription. Unfortunately, record of the number of administered doses is not done systematically.

Periodic surveys to estimate the uptake rates of seasonal influenza vaccine could help to monitor success of national vaccination programs, to provide important information on the number of vaccine doses administered, rather than simply purchased, and to assess the degree of vaccination awareness in the surveyed population [14, 15]. Identifying factors associated with influenza vaccination behaviors among older persons may help to better design interventions to improve vaccine coverage [16]. This study aimed to evaluate influenza vaccine uptake in elderly persons with chronic disease(s) in Tunisia during the 2018–2019 influenza season, and to describe their knowledge and attitudes towards influenza and influenza vaccination. Our ultimate objective is to identify predictors of seasonal influenza vaccine uptake and related barriers in order to reduce the burden of influenza among this high risk group.

## Methods

### Study design

A cross-sectional study was conducted among elderly persons with chronic disease(s), between March and June 2019, who were attending primary and secondary heath care facilities.

### Study population

The study population included: persons aged 60 years and over who sought care at public primary health centers, regional hospitals, or district hospitals, and who had at least one of the following chronic diseases/conditions: respiratory disease (chronic obstructive pulmonary diseases including asthma or other chronic pulmonary diseases); cardiac disease; diabetes; chronic renal failure requiring dialysis; cirrhosis; morbid obesity; and immunocompromising conditions (including patients undergoing chemotherapy or chronic corticosteroid therapy, and transplant patients taking immunosuppressive drugs). We conducted our study among this population since they represent a priority target group for influenza vaccination in Tunisia compared to healthy elderly. Individuals who did not provide written informed consent and those with cognitive disorders were not included in this survey.

### Sample size and sampling procedure

In order to collect a representative sample of the target population, we estimated the sample size using Slovin’s formula. We took into consideration an expected size of elderly persons with chronic diseases (across both primary and secondary health centers) of between 10,000 and 100,000, a precision of 0.05, a design effect of 2.5, and a non-response rate equal to 20% leading to a sample size of ≈ 1200 individuals. We choose a self-weighted two-stage sampling scheme to ensure geographic representativeness at the country level. First a stratified sampling was performed based on the three Tunisian regions (North, Center and South). Eight out of the 24 Tunisian governorates were randomly selected: four in the north (Ariana, Ben Arous, Bizerte, Siliana), three in the center (Kairouan, Mahdia, Sousse), and one in the south (Gafsa). Following this, we proceed in each gouvernorate to selection by simple random sampling of health care facilities offering a consultation for persons with chronic diseases. Since the study was carried out on working days and that the days of consultation for chronic disease patients differ according to the health care centers; the selection of centers was made as the survey evolved. Every day a random sampling of one facility from the list of the health care centers offering consultation to chronically ill patients that day was performed. Each center already selected will no longer be selected afterwards. The recruitment of eligible persons was stopped after the achievement of the target sample size.

It should be noted that the calculated sample size was distributed according to the repartition of Tunisian elderly between the three Tunisian regions, to the weight of each governorate in the corresponding region and to the weight of the area of residence in each of the selected governorates.

### Data collection

All illegible patients who were present during the visits of investigators to the selected healthcare facilities were approached to participate in the survey and informed about the aims of the study and the anonymity of participants. Those who accepted to participate signed an informed consent. A face to face standardized questionnaire was administered in Arabic to participants by trained investigators (medical doctors and paramedics).

The questionnaire was composed of three sections (Additional file 1). The first one included questions about participant’s medical history. The second contained questions related to practices, general knowledge and attitudes regarding influenza and influenza vaccine. Open ended questions were used to assess reasons of influenza vaccination acceptance and refusal, in addition to trusted sources of information about influenza vaccine. General statements regarding influenza and influenza vaccine were assessed using a 5-point Likert type items (1: Strongly Agree, 2: Agree, 3: Neutral, 4: Disagree, 5: Strongly Disagree). Before the beginning of the study, a meeting was conducted with all field investigators to assess the face to face validity of the questionnaire mainly questions wording and appropriateness of the survey instrument and to train investigators. To evaluate the understanding of questions, a pilot study was conducted in a primary health care facility in the governorate of Manouba which is located in Northern Tunisia but not selected in our survey. Thirty elderly with chronic diseases were interviewed during the pilot study. Collected data were not included in the final analysis.

In the present study, respondents did not receive any gift or monetary compensation for their participation.

### Data analysis

Statistical analysis included a descriptive analysis where qualitative variables were summarized as numbers and percentages. The relationship between willingness to receive influenza vaccine and each of the explanatory categorical variables was determined by Fisher exact χ^2^ test and crude odds ratios (COR). Independent variables with a *p*-value ≤0.25 in the bivariate analysis were introduced in the multivariate model using a backward stepwise logistic regression. To measure the strength of association between explanatory variables and willingness to receive the vaccine and control for potential effect modifiers, adjusted odds ratios (AOR) were estimated by multivariate analyses. Missing and “I don’t know” or “I don’t remember” responses were excluded from these analyses. Comparisons of attitudes and general statements regarding influenza and influenza vaccine were performed by Fisher exact χ^2^ test between participants who wanted to receive the vaccine and those who refused to be vaccinated. For all analyses, two-tailed tests were used and a *p*-value ≤0.05 was considered significant.

## Results

The study was conducted in 100 health care facilities: 47 in Northern Tunisia, 42 in the Center and 11 in the South. Overall, 1318 elderly persons with chronic diseases were approached to participate to the survey. Among them 1235 accepted to participate to the survey and were interviewed, giving a response rate of 93.7%. Only 1191 questionnaire were considered eligible for analysis. The 44 excluded forms were either missing important data or related to individuals non-complying with the inclusion criteria (some respondents were under 60 years of age or had isolated high blood pressure).

Nearly two-thirds of the participants were female and from urban areas. The age of participants ranged from 60 to 99 years; over half were aged 60–69. About 87% of patients had a primary school level education or less, and less than 4% of participants are still working (Table 1). Over 80% of participants were diabetic, and one-quarter had heart disease (Table 1).

**Table 1:** Main characteristics of participants

Variables	n (%)
**Socio-demographic Characteristics**	
**Area of residence (*n*** **= 1191)**	
Urban	797 (66.9)
Rural	394 (33.1)
**Gender (*n*** **= 1190)**	
Men	429 (36.1)
Women	761 (63.9)
**Age class (years) (*n*** **= 1191)**	
60–69	654 (54.9)
70–79	414 (34.8)
> =80	123 (10.3)
**Level of education (*n*** **= 1190)**	
Primary level or less	1034 (86.9)
Secondary level or higher	156 (13.1)
**Occupational status (*n*** **= 1187)**	
Work	41 (3.5)
Retired/ Do not work	1146 (96.5)
**Chronic disease (*n*** **= 1191)**	
**Asthma**	93 (7.8)
**Other chronic pulmonary disease**	84 (7.1)
**Heart disease**	302 (25.4)
**Diabetes**	988 (83.0)
**Kidney failure at the hemodialysis stage**	14 (1.2)
**Hepatic cirrhosis**	6 (0.5)
**Morbid obesity**	24 (2.0)
**Vaccination against influenza at least once in the past**	
Yes	415 (34.8)
No	773 (64.9)
I don’t remember	3 (0.3)
**Vaccination against influenza in 2018–2019 season**	
Yes	231 (19.4)
No	960 (80.6)
**Willingness to receive influenza vaccine next season**	
Yes	771 (64.7)
No	331 (27.8)
I don’t know	89 (7.5)

Among respondents, 70.1% [67.4–72.9] were aware about the existence of a vaccine against influenza. Over one-third of participants (34.8% [32.2–37.6]) took the influenza vaccine at least once in the past and 19.4% [17.1–21.9] received this vaccine during the 2018–2019 influenza season, while 64.7% [61.9–67.3] of surveyed persons expressed their willingness to be vaccinated (Table 1).

Compared to participants who refused vaccination, persons who were willing to receive influenza vaccine the next season were more convinced that influenza was more dangerous for elderly persons (88.5% vs. 77.3%, *p* < 0.001) and for persons with chronic diseases (88.4% vs. 81.0%, *p* = 0.004) (Table 2). Participants willing to receive the vaccine were significantly more persuaded by the effectiveness of the vaccine and perceived less its side effects for elderly persons and those with chronic disease (*p* < 0.001).

**Table 2:** Knowledge and attitudes towards general statements about influenza and influenza vaccine stratified by vaccination intention

Statements about influenza and influenza vaccine^**a**^	Willing to receive vaccine		Not willing to receive vaccine		***P*** value
	N^**b**^	Agree^**d**^ n(%)	N^**c**^	Agree^**d**^ n (%)	
**Influenza does NOT cause a lot of illness in Tunisia**	657	128(19.5)	248	42(16.9)	0.382
**People who have influenza are never sick enough to be admitted to the hospital**	656	118(18.0)	247	52(21.1)	0.294
**Influenza is more dangerous for elderly persons**	655	580(88.5)	247	191(77.3)	< 0.001
**Influenza is more dangerous for persons with chronic diseases**	656	580(88.4)	247	200(81.0)	0.004
**Influenza vaccine can make someone sick with influenza**	571	96(16.8)	215	64(29.8)	< 0.001
**Influenza vaccine is recommended for elderly persons**	572	478(83.7)	215	127(59.1)	< 0.001
**Influenza vaccine helps protect elderly persons against influenza**	572	483(84.4)	215	126(58.6)	< 0.001
**Influenza can be not safe for elderly persons**	572	57(10.0)	216	45(20.8)	< 0.001
**Influenza vaccine is recommended for persons with chronic disease**	570	454(79.6)	216	115(53.2)	< 0.001
**Influenza vaccine helps protect persons with chronic disease against influenza**	570	463(81.2)	215	120(55.8)	< 0.001
**Influenza can be not safe for persons with chronic diseases**	571	58(10.2)	215	40(18.6)	0.001
**influenza vaccine is recommended annually for elderly persons with chronic disease**	572	378(66.1)	216	115(53.2)	0.001

^a^Statements about influenza concern only participants who had knowledge about influenza while statements about influenza concern only participants who know that there exist a vaccine

^b^The number of responses for each statement among participants that indicated a willingness to receive influenza vaccine

^c^The number of responses for each statement among participants that indicated unwillingness to receive influenza vaccine

^d^“strongly agree” and “agree” were gathered in “agree” while “strongly disagree”, “disagree”, “neutral” and I don’t know” were gathered together in “other attitude”

Doctors were by far the most trusted source for information regarding influenza vaccine (91.5%). Only 2.9% of surveyed persons trusted the media as reliable source of information. No participants cited the internet as trusted source for influenza vaccine-related information. The two main reasons that may lead the elderly to accept influenza vaccination were a doctor’s recommendation (41.1%) and protection against contracting influenza (39.6%). The two main reasons that may lead to refusal of vaccination among the elderly were concerns that the vaccine could cause side effects (71.5%) and a belief that the vaccine could be ineffective (33.9%) (Table 3).

**Table 3:** Most trusted sources and top reasons for accepting and refusing to receive influenza vaccine

Reasons	n (%)
**Trusted sources for information about influenza vaccine (*n*** **= 1120)**^**a**^	
Doctor	1025(91.5)
Pharmacist	197(17.6)
Other healthcare professional	158(14.1)
Family member	95(8.5)
Media	33(2.9)
Internet	0(0.0)
**Reasons that may lead to accept influenza vaccination (*n*** **= 904)**^**b**^	
If the doctor recommended the vaccine for me	373(41.1)
To protect myself against influenza	358(39.6)
To not contract a severe form of influenza	66(7.3)
If the vaccine is effective	54(5.9)
If the vaccine is free or the cost is acceptable	52(5.7)
Because I have a chronic disease/ because I am an elderly person	47(5.2)
To protect my family against influenza	40(4.4)
If I get influenza frequently/ if I contract a severe form of influenza	34(3.8)
If there is an influenza epidemic	34(3.8)
To not decompensate my chronic disease	27(2.9)
**Reasons that may lead to refuse influenza vaccination (*n*** **= 661)**^**b**^	
Concerns that the vaccine could cause side effects	473(71.5)
Belief that the vaccine is not effective	224(33.9)
Belief that a disease like influenza is not dangerous	101(15.3)
Belief that it is better to suffer the natural disease than to be vaccinated	76(11.5)
Household member does not authorize me to get the vaccine	43(6.5)
If Doctor do not recommend to receive the vaccine	40(6.0)
Financial reasons/ cost of the vaccine	34(5.1)
Belief that the vaccine could cause influenza	13(1.9)
Religious reasons	5(0.7)
Ethical, or moral reasons	1(0.1)

^a^Respondents indicated “I don’t know” or “No one” answers or skipped the question are not included in Table 3 calculations

^b^Respondents indicated “I don’t know” or “No reason” answers or skipped the question are not included in Table 3 calculations

In the bivariate analysis, willingness to receive influenza vaccine among the elderly was not significantly associated to socio-demographic factors. Participants with chronic pulmonary diseases were 2.2 times more likely to be willing to receive the influenza vaccine (*p* = 0.008), while surprisingly those with heart disease were less likely to accept vaccine (COR = 0.57 [0.43–0.73]). Persons who had knowledge about influenza, those who are aware about the existence of a vaccine and those who knew someone who was severely ill with influenza were significantly more willing to receive the influenza vaccine (COR = 1.95 [1.42–2.68], COR = 1.47 [1.11–1.94] and COR = 1.45 [1.08–1.94] respectively). Participants who estimated that they received enough information about the safety of influenza vaccine and vaccines in general showed higher willingness to receive the vaccine (COR = 2.29 [1.40–3.74] and COR = 5.62 [3.34–9.46] respectively). Receiving an influenza vaccine recommendation and trusting the advice of healthcare providers were positively associated with willingness to receive vaccine among elderly persons (COR = 4.11 [3.02–5.60] and COR = 7.60 [4.12–14.03] respectively). The odds of willing to receive influenza vaccine among vaccinated persons during the 2018–2019 influenza season were 45.3 times higher compared to unvaccinated ones (*p* < 0.001).

Multiple logistic regression analysis revealed that willingness to receive influenza vaccination was more likely among participants with chronic pulmonary disease (AOR = 2.63 [1.15–6.03]), those suffering from diabetes (AOR = 1.67 [1.05–2.65]), those who received the influenza vaccine during the 2018–2019 season (AOR = 16.53 [3.77–72.45]), those who felt they got enough information about vaccines and their safety (AOR = 2.91 [1.59–5.34]), and those who trusted the advice of their health care provider (AOR = 4.49 [2.19–9.24]).

## Discussion

We examined the influenza vaccination rate among 1191 Tunisian elderly persons with chronic disease(s) during the 2018–2019 influenza season and factors influencing their intention to receive the vaccine. About one-fifth of participants were vaccinated during the 2018–2019 influenza season, while nearly two-thirds expressed willingness to receive influenza vaccination.

Vaccine uptake among the study population was 19.4%, far below the 75% recommended by the WHO. It is possible that the overall coverage of influenza vaccine uptake among the Tunisian older population (including healthy persons and those with underlying conditions) was lower than the rate found in our study population. In fact, the presence of comorbidities has been identified in several studies as a predictor of influenza vaccination in the elderly [15–18]. Older persons with chronic medical conditions may be more motivated to receive vaccination because they perceived themselves to be at higher risk for influenza complications [15, 19]. Moreover, health professionals tend to stress the importance of vaccination among this high-risk group [15, 17, 19].

The vaccine coverage estimated in our study was lower than that found among elderly in some member states in the Mediterranean region of the Organization for Economic Co-operation and Development (OECD), including Portugal (60.8%), Spain (50.7%), Italy (52.7%), France (49.7%), and Greece (48.9%) [20]. In contrast, the uptake in Tunisia was higher than that observed in Slovenia (11.8%) and Turkey (7.0%) [20]. Only Korea (82.7%) and Mexico (82.3%), two member states of the OECD, have reached the coverage recommended by the WHO [20]. In Africa, very little data is available regarding influenza vaccine uptake among the elderly, despite the sharp increase in the impact of influenza due to the burden of other diseases such as HIV/AIDS and tuberculosis [21]. A recent study among South African private health insurance scheme members showed that the vaccination uptake in persons older than 65 years was 18.5% [21]. Similarly, vaccine uptake rates reported in previous studies among patients with chronic conditions have varied. In a study conducted among patients with chronic diseases visiting an Italian out-patient clinic, Napolitano et al. found that nearly half of participants were vaccinated against influenza in 2018–2019 [22]. However, Ye et al. and Mohr et al., [23, 24] reported that only 7.8% of diabetic patients in china and less than 30% of patients with chronic pulmonary diseases in Germany received influenza vaccine respectively.

While the vaccination uptake rate was low among our study population, nearly two-thirds of participants expressed willingness to receive the influenza vaccine. Most previous studies examined factors associated with previous influenza vaccine uptake, but this study’s main interest was in the factors associated with willingness in regard to future vaccination. In fact, even if influenza vaccine uptake could be predictive of future uptake, receipt of vaccine may vary from season to season [7, 25–27]. According to Zimmerman et al. [28], intention was “the strongest predictor of behavior”, however the environment and the cues for action such as the medical advice are also strong motivators as shown in our study.

We found that willingness to receive influenza vaccine among the elderly was not influenced by socio-demographic factors. Compared to persons with other diseases, people suffering from chronic pulmonary disease and those with diabetes were more willing to be vaccinated, which is consistent with two previous studies that found older persons with pulmonary diseases are more likely to be vaccinated [29, 30]. These groups may have higher perceived severity when they contract the influenza virus, since it is a respiratory infection that could decompensate chronic pulmonary diseases or lead to severe acute respiratory distress when the infected is immunocompromised. There remains a need to improve knowledge about influenza vaccination in the older population and to inform elderly about chronic conditions that can increase the morbidity and mortality tolls of influenza. In agreement with previous studies, persons willing to receive influenza vaccine were more convinced of the danger of influenza for elderly persons and for persons with chronic diseases. They were also significantly more persuaded by the effectiveness of the vaccine, and perceived less its side effects for elderly persons compared to those who refused vaccination [16, 31]. In our study, reasons reported as discouraging the influenza vaccine uptake were, mainly concerns that the vaccine could cause side effects (71.5%) and beliefs of its ineffectiveness (33.9%). These findings, corroborates the conclusions of previous studies [2, 18]. It is important to correct misconceptions about adverse events and efficiency of the vaccine [1]. In fact, influenza vaccines are generally well tolerated and safe among elderly [6] and can even confer cross-protection when the circulating strains are not well matching the vaccine strains [32].

Our study is consistent with previous findings that demonstrated that willingness to be vaccinated was strongly associated with former vaccination status [23, 26, 28, 31, 33–36]. Indeed, positive experience with the vaccine may lead to perception of higher benefits and fewer barriers to vaccination [36], making those who received the vaccine more likely to be regularly compliant [33, 34]. In fact, once individuals have adopted a behavior, they are likely to change their beliefs to be in agreement with this behavior [37]. Thus, campaigns promoting influenza vaccination among the elderly may have a snowball effect, inducing a cumulative increase in vaccination rates every year [35].

The multivariate analysis showed that trusting the advice of heath care providers was significantly associated with willingness to receive vaccination among elderly persons. We found the main reason for accepting influenza vaccine was receiving a doctor’s recommendation (41.1%) as well. The most trusted sources for information about influenza vaccine were doctors (91.5%), followed by pharmacists (17.6%), and then other healthcare professionals (14.1%). These findings highlighted the important role that physicians could play in improving influenza vaccination uptake among elderly people with chronic disease(s). They are consistent with previous studies showing that recommendations from health care professionals are one of the strongest facilitators for influenza vaccination [1, 16, 23, 34]. Physicians in particular have a crucial role in educating their patients to adopt relevant attitudes and practices toward influenza vaccination [1]. Paradoxically, according to a systematic literature review, several studies reported that many physicians do not recommend the vaccine to their patients [34]. Potential explanations were that doctors cover a wide range of topics during consultations, underestimating the key influence vaccines can play, etc. [34, 35, 38]. A study conducted in Taiwan suggested that physicians give more importance to treatment than to prevention of diseases, and that some doctors may fear further burdening an already weakened immune system in older patients with chronic diseases [35]. This confirms the need to promote the comprehensive approach in the medical practice including, promotion, prevention and restauration of health. Our study advocates for targeting the health care professionals, who should be aware of their crucial role in promoting influenza vaccination among their patients as demonstrated elsewhere [39, 40].

Our study is one of the first on influenza vaccination among elderly with chronic diseases, in Tunisia. Our findings could be of great importance in the current context of COVID-19 pandemic since some factors influencing the attitudes of elderly people towards influenza vaccine could also determine their decisions regarding COVID-19 vaccination which is also recommended for this population group. This study had several limitations. First we did not include those who are seeking care at the university hospitals or in the private sector. These patients might have more severe stages of chronic diseases which might affect the generalizability of our findings, as their knowledge, attitudes, and practices may differ from those in our survey. Second, data about vaccine uptake were self-reported and not validated with medical records; therefore findings might be subject to recall biases [1, 7, 16–18, 31]. We believe that the exclusion of persons with even mild cognitive disorders reduces this potential bias. Unfortunately, medical validation of influenza vaccine uptake was not always possible. Third, as many elderly participants were illiterate we did not use a self-administered questionnaire, which may lead to a social desirability bias [33]. Finally, the cross-sectional design of our study did not allow for inferring causal relationships between dependent and independents variables [19, 26].

## Conclusions

This study revealed a low coverage of influenza vaccine uptake among elderly with chronic diseases and identified the main barriers and predictors for influenza vaccination in Tunisia among this target group. Results further suggested that influenza vaccine promotion campaigns should involve more health care professionals. In fact, promoting a comprehensive practice of care including vaccination for all age groups among health care providers can increase the rate of influenza vaccine uptake through time. A training program on influenza and influenza vaccine might be a good strategy to encourage them promoting this preventive measure.

## Supplementary Information

**Additional file 1.** Questionnaire.

## Data Availability

The datasets used during the current study are available from the corresponding author on reasonable request.

## Abbreviations

AIDS: Acquired Immunodeficiency Syndrome
AOR: Adjusted Odds Ratio
COR: Crude Odds Ratio
COVID-19: Coronavirus Disease 2019
HIV: Human Immunodeficiency Virus
OECD: Organization for Economic Co-operation and Development
WHA: World Health Assembly
WHO: World Health Organization
