# Supplementary material for: Influenza vaccination knowledge, attitudes, and practices among Tunisian elderly with chronic diseases
Source: BMC Geriatr. 2021 Dec 15;21:700. doi: 10.1186/s12877-021-02667-z (PMC8672335; doi:10.1186/s12877-021-02667-z)
Supplement: Supplementary file 1 — Additional file 1. Questionnaire. [file 12877_2021_2667_MOESM1_ESM.docx]

Additional file 1: questionnaire

**Knowledge, Attitudes, and Practice Survey Questionnaire: Elderly with chronic disease**

**ID participant** : _________________

**Name of interviewer**: ______________________

**Governorate :** ________________________ **District :** __________________________________

**Health Facility Name:** __________________________________

**Participant’s area of residence : urban/rural**

1. **Health history**
2. **Among the following diseases, which one(s) do you present? How long have you been affected by this (these) disease(s)**

|  | Yes/No | duration |
| --- | --- | --- |
| Asthma |  |  |
| Other pulmonary disease |  |  |
| Heart disease |  |  |
| diabetes |  |  |
| Chronic renal insufficiency on dialysis |  |  |
| Cirrhosis |  |  |
| Morbid obesity |  |  |
| Other(s) |  |  |

1. **Do you have a condition inducing immunocompetency disorder:**
2. ☐No
3. ☐Chemotherapy
4. ☐Organ transplantation
5. ☐Chronic corticosteroid therapy
6. **Knowledge, attitudes and practice regarding influenza and influenza vaccine**
7. **Have you ever heard of seasonal influenza or the flu before?**
8. ☐ Yes
9. ☐ No **(skip to question 16)**
10. **I will give you 4 general statements about seasonal flu, please State your point of view regarding them.**

|  | **Strongly Agree** | **Agree** | **Neutral** | **Disagree** | **Strongly Disagree** | **I don’t know** |
| --- | --- | --- | --- | --- | --- | --- |
| a. Influenza does NOT cause a lot of illness in Tunisia |  |  |  |  |  |  |
| b. People who have influenza are never sick enough to be admitted to the hospital |  |  |  |  |  |  |
| c. Influenza is more dangerous for persons with chronic diseases |  |  |  |  |  |  |
| d. Influenza is more dangerous for elderly persons |  |  |  |  |  |  |

1. **Do you know anyone who has been severely ill with influenza?**
2. ☐ Yes
3. ☐ No
4. ☐ I don’t remember
5. **Have you ever heard of the vaccine against influenza?**
6. ☐ Yes
7. ☐ No **(skip to question 16)**
8. **Did you receive the influenza vaccine at least once in the past?**

- 1. ☐ Yes
  2. ☐ No **(skip to question 10)**
  3. ☐I don’t **(skip to question 10)**

1. **Did you receive the influenza vaccine during 2018-2019 influenza season?**
2. ☐ Yes
3. ☐ No
4. ☐ I don’t remember
5. **How many times did you receive influenza vaccine in the past 5 years?**
6. ☐Never
7. ☐Once
8. ☐2 to 4 times
9. ☐5 times
10. ☐I don’t remember
11. **Has anyone recommended you receive the influenza vaccine?**
12. ☐ Yes
13. ☐ No
14. ☐ I don’t remember
15. **Has anyone discouraged you from receiving the influenza vaccine?**
16. ☐ Yes
17. ☐ No
18. ☐ I don’t remember
19. **Have you heard or read reports in the media or social media that would make you hesitate to be vaccinated with influenza vaccine?**
20. ☐ Yes
21. ☐ No
22. ☐ I don’t know
23. **Do you know anyone who has ever had a bad reaction to influenza vaccine, which may discourage you from getting vaccinated?**
24. ☐ Yes
25. ☐ No
26. ☐ I don’t know
27. **Do you feel you get enough information about influenza vaccines and their safety?**
28. ☐ Yes
29. ☐ No
30. ☐ I don’t know
31. **I will give you 8 general statements about influenza vaccine, please state your point of view regarding them.**

|  | **Strongly Agree** | **Agree** | **Neutral** | **Disagree** | **Strongly Disagree** | **I don’t know** |
| --- | --- | --- | --- | --- | --- | --- |
| a. Influenza vaccine can make someone sick with influenza |  |  |  |  |  |  |
| b. Influenza vaccine is recommended for elderly persons |  |  |  |  |  |  |
| c. Influenza vaccine helps protect elderly persons against influenza |  |  |  |  |  |  |
| d. Influenza can be not safe for elderly persons |  |  |  |  |  |  |
| e. Influenza vaccine is recommended for persons with chronic disease |  |  |  |  |  |  |
| f. Influenza vaccine helps protect persons with chronic disease against influenza |  |  |  |  |  |  |
| g. Influenza can be not safe for persons with chronic diseases |  |  |  |  |  |  |
| h. influenza vaccine is recommended annually for elderly persons with chronic disease |  |  |  |  |  |  |

1. **Would you want to receive the vaccine against influenza?**
2. ☐ Yes
3. ☐ No
4. ☐ I don’t know
5. **What are the top three reasons that would persuade you to take the influenza vaccine?**
   - - 1. ____________________________________________
       2. ____________________________________________
       3. ____________________________________________
6. **What are the top three reasons that would discourage you from taking the influenza vaccine?**
   - - 1. ____________________________________________
       2. ____________________________________________
       3. ____________________________________________
7. **Who (or what source) would you trust the most to give you the most accurate information about influenza vaccines? (Open ended question)**

a. ☐Doctor

b. ☐Pharmacist

c. ☐Other healthcare worker : ________________________

d. ☐Social Worker

e. ☐A Familly member :________________________

f. ☐Media

g. ☐ Internet

h. ☐ Other: _____________________________________

1. ☐ I don’t know
2. **In general, do you feel you get enough information about vaccines and their safety?**
3. ☐ Yes
4. ☐ No
5. ☐ I don’t know
6. **In general, do you trust the advice of your health care provider (Doctor/Nurse)?**
7. ☐ Yes
8. ☐ No
9. ☐ I don’t know
10. **General and Demographic Questions**
11. **Gender :**
    1. ☐ male
    2. ☐ female
12. **How old are you?** ……………….

**Date of birth :....../……/………….**

1. **What is your marital status?**
   1. ☐ Single / never married
   2. ☐ Married
   3. ☐ Divorced / separated
   4. ☐ Widowed
   5. ☐ Other: ________________________________
   6. ☐I don’t want to answer
2. **What is your education level?**
   1. ☐ Primary level or less
   2. ☐ Secondary level or higher
   3. ☐ I don’t want to answer
3. **What is your Occupational status ?**
   1. ☐ I’m retired/ I don’t work
   2. ☐ I work
4. **What comments would you like to make about your concerns about influenza and influenza vaccine for elderly person with chronic diseases in Tunisia?**

**______________________________________________________________________________________________________________________________________________________________________________________________________________________________________________________________________________________________________________________________________________________________________________________________________**

**Thank you for answering our questions.**
